# Cerebellar Abnormalities Contribute to Disability Including Cognitive Impairment in Multiple Sclerosis

**DOI:** 10.1371/journal.pone.0086916

**Published:** 2014-01-22

**Authors:** Katrin Weier, Iris K. Penner, Stefano Magon, Michael Amann, Yvonne Naegelin, Michaela Andelova, Tobias Derfuss, Christoph Stippich, Ernst-Wilhelm Radue, Ludwig Kappos, Till Sprenger

**Affiliations:** 1 Department of Neurology, University Hospital Basel, Basel, Switzerland; 2 Department of Cognitive Psychology and Methodology, University of Basel, Basel, Switzerland; 3 Department of Radiology, Division of Neuroradiology, University Hospital Basel, Basel, Switzerland; 4 Medical Image Analysis Center (MIAC) AG, University Hospital Basel, Basel, Switzerland; University of Missouri-Kansas City, United States of America

## Abstract

The cerebellum is known to be involved not only in motor but also cognitive and affective processes. Structural changes in the cerebellum in relation to cognitive dysfunction are an emerging topic in the field of neuro-psychiatric disorders. In Multiple Sclerosis (MS) cerebellar motor and cognitive dysfunction occur in parallel, early in the onset of the disease, and the cerebellum is one of the predilection sites of atrophy. This study is aimed at determining the relationship between cerebellar volumes, clinical cerebellar signs, cognitive functioning and fatigue in MS. Cerebellar volumetry was conducted using T1-weighted MPRAGE magnetic resonance imaging of 172 MS patients. All patients underwent a clinical and brief neuropsychological assessment (information processing speed, working memory), including fatigue testing. Patients with and without cerebellar signs differed significantly regarding normalized cerebellar total volume (nTCV), normalized brain volume (nBV) and whole brain T2 lesion volume (LV). Patients with cerebellar dysfunction likewise performed worse in cognitive tests. A regression analysis indicated that age and nTCV explained 26.3% of the variance in SDMT (symbol digit modalities test) performance. However, only age, T2 LV and nBV remained predictors in the full model (r^2^ = 0.36). The full model for the prediction of PASAT (Paced Auditory Serial Addition Test) scores (r^2^ = 0.23) included age, cerebellar and T2 LV. In the case of fatigue, only age and nBV (r^2^ = 0.17) emerged as significant predictors. These data support the view that cerebellar abnormalities contribute to disability, including cognitive impairment in MS. However, this contribution does not seem to be independent of, and may even be dominated by wider spread MS pathology as reflected by nBV and T2 LV.

## Introduction

Multiple Sclerosis (MS) is a chronic demyelinating disease of the central nervous system. Recent studies investigating atrophy in MS support the view that neurodegeneration contributes significantly to the accrual of disability [1]. So far, most neuroimaging and neuropathological studies focused primarily on atrophy of the cerebral hemispheres as reliable measurement of atrophy in structures, such as the brainstem or cerebellum, is technically challenging [2], [3]. There is some evidence that the cerebellum is supporting non-motor functions such as cognition, mood and behaviour [4] and that cerebellar dysfunction may play a role in the development of disability [5], [6]. Several studies have pointed out the relevance of connecting loops between the cerebellum and the frontal, superior temporal, limbic and posterior parietal cortex in cognitive processing [7]. Schmahmann and Sherman [8] have previously described the so-called “cerebellar cognitive affective syndrome” with deficits in executive functioning, language, and spatial cognition, as well as, behavioural changes in patients who had cerebellar lesions due to stroke or hereditary disorders (e.g. cerebellar ataxias). In MS patients, both cerebellar signs and cognitive dysfunction are significant contributors to the development of disability and often progress despite disease-modifying treatment [9], [10]. It is characteristic for MS that instead of a global cognitive decline, specific cognitive domains are affected [11]. Most frequently, memory, attention, information processing speed, abstract/conceptual reasoning, and visuospatial skills are affected while primary language skills, long-term memory and verbal intelligence appear to be less impaired [12], [13]. Recent data have suggested that whole brain or grey matter atrophy rather than lesion burden may predict cognitive decline [14], [15]. Most of these studies focused on global rather than regional atrophy. Both, Calabrese et al. [16] and Anderson et al. [17] have shown significant reductions of cerebellar grey matter in MS patients. However, both of these studies focused only on correlations between cerebellar volume loss and cerebellar motor function.

To the best of our knowledge, no data have been published focusing on the relation between cerebellar atrophy and cognitive deficits in MS. The aim of this study was to test whether performance in the cognitive domains of information processing speed and working memory relate to cerebellar volume in a large cohort of MS patients.

## Materials and Methods

### Ethics Statement

Written informed consent was obtained from each patient before the examination. Ethics approval was provided by the “Ethikkommission beider Basel” (EKBB).

### Patients

We analysed the data of 172 patients (112 women, mean age 48.1±10.9 years, range 24–70 years) taking part in an on going cohort study on the genotype-phenotype characterization of MS. Four patients (one woman) had a clinically isolated syndrome, 122 patients (89 women) had relapsing-remitting (RRMS) MS, 35 patients (16 woman) were secondary progressive (SPMS) and 11 patients (six women) had been diagnosed with primary progressive MS (PPMS).

### Neuropsychological and Clinical Assessment

All patients underwent a medical and neurological examination, including a structured assessment of the Expanded Disability Status Scale [18] (EDSS; incl. the cerebellar functional system score (cFSS)) by a trained rater, and the nine hole peg test [19] (9-HPT). The neuropsychological assessment included the Symbol Digit Modalities Test [20] (SDMT) and the Paced Auditory Serial Addition Test [19] (PASAT 3). The patients completed self-assessment questionnaires for fatigue [21] (FSMC = Fatigue Scale for Motor and Cognitive Functions) and handedness [22] (Edinburgh Handedness Inventory).

### MRI

Cerebellar segmentation and volumetry was performed on high resolution T1 weighted MPRAGE images acquired in sagittal plane (TR/TI/TE = 2080/1100/3.0 ms; α = 15°, 160 slices, isotropic voxel of 1×1×1 mm). Axial 3 mm proton density-weighted (PDw) and T2-weighted (T2w) images were acquired to determine the cerebral and cerebellar lesion load (double spin echo: TR/TE1/TE2 = 3980/14/108 ms; 40 slices with an in-plane resolution of 1×1 mm). MRI scanning was performed at the Department of Radiology, Division of Neuroradiology, University Hospital Basel, Switzerland using a 1.5 T Magnetom Avanto scanner (Siemens Medical Solutions, Erlangen, Germany).

### MRI Data Postprocessing

Total cerebellar volume (TCV), cerebellar grey matter volume (CGV) and white matter volume (CWV) were determined semi-automatically using the 3D T1w images with ECCET [23] (www.eccet.de). Total normalized brain volume (nBV) was assessed using SIENAX [24]. The SIENAX normalization factor was used for normalizing the cerebellar volumes in order to correct for head size. All analyses were performed on the normalized volumes. Additional conventional MRI measures such as total T2w and T1w lesion volumes (LV) were also assessed.

### Statistical Analyses

Patients with and without cerebellar dysfunction were grouped according to the cerebellar functional system score (0 or >0). Between group comparisons for cerebellar and whole brain volumes, 9-HPT, as well as, cognitive and fatigue testing were performed using ANCOVA with age and sex as covariates. Non-normally distributed data were normalized using a logarithmic transformation for PASAT and an inverse transformation for 9-HPT. In case of the PASAT, the base-10 logarithm was applied to the difference between the maximum value plus one and the subject performance in order to flip the distribution. Rank analysis of covariance was used for cerebellar T1 and whole brain T2 LV [25]. In the case of PASAT and SDMT, z-scores were computed as in Scherer and colleagues [26]. Subjects with extreme scores (z-scores<−4 for PASAT and SDMT; two outliers each) were excluded as proposed by Hill et. al. [27].

Hierarchical multiple linear regression (MLR) analyses, including three blocks, were performed to investigate the relation between cerebellar and whole brain metrics, and cognitive functioning (SDMT, PASAT), as well as, fatigue (FSMC sum (FSMC-S) score). Demographical data (age and gender) were entered into the first block of the model and maintained in order to take them into account in all subsequent blocks. In the second block, cerebellar metrics (nTCV, cerebellar T1 LV) were included stepwise in order to select for significance (variable entry at P<0.05, variable removal P>0.10). With the same stepwise approach, whole brain metrics (nBV, whole brain T2 LV) were included in the third block. For all MLR models the regression analysis assumptions were tested. All statistical analyses were performed using SPSS 20 (IBM, New York, USA). A p-value of <0.05 was used for statistical thresholding.

## Results

### Demographic Data (Table 1)

Patients had a mean disease duration of 16.2±9.5 years (range 3–50) and a median EDSS of 3.0 (range 0–7). The median cerebellar functional system score (FSS) was 2.0 (range 0–4) and the mean performance time for the 9-HPT of the dominant hand was 23±19.7 seconds (range 15–66). Seventy percent (120/172) of patients showed cerebellar signs, as defined by a cerebellar FSS of >0. Patients with cerebellar signs were older (t(170) = −5.71; p<0.001), had a significantly longer disease duration (t(148.7) = −5.2, p<0.001) and had higher EDSS (U = 603.5; p<0.001) than patients without cerebellar signs. No confounding co-morbidities, possibly affecting the cerebellum, were noted.

**Table 1:** Demographic and clinical data.

	Total cohort (n = 172)	Patients without cerebellar signs (n = 52)	Patients with cerebellar signs^2^ (n = 120)
	mean ±SD (range)	mean ±SD (range)	mean ±SD (range)
	^+^median (range)	^+^median (range)	^+^median (range)
**Age (years)**	48.1±10.9 (23–70)	41.5±10.1 (23–67)	51±9.9 (29–70)***
**Sex (f:m)**	112∶ 60	40∶12	72∶ 48*
**Disease duration (years)**	16.2±9.5	11.7±6.2	18.2±10***
**Disease course**			
CIS (n)	4	2	2
RRMS (n)	122	50	72
SPMS (n)	35	0	35
PPMS (n)	11	0	11
**EDSS^+^**	3.0 (0–7.5)	1.5 (0–7.5)	4.0 (1–7.5)***
**cerebellar FSS^+^**	2.0 (0–4)	0	2.0 (1–4)
**motor FSS^+^**	2.0 (0–4)	1.0 (1–4)	2.0 (1–4)
**9-HPT dominant hand (seconds)**	23±19.7 (15–66)	18±2.2 (15–25)	25±10.7 (15–66)***
**SDMT (z-score)**	−1.11±1.9 (−3.65 – 4.)	−0.41±1.28 (−2.9–4)	−1.42±1.01 (−3.65–1.25)***
% of impaired patients^1^	30	10	42
**PASAT (z-score)**	−0.68±1.04 (−3.91 – 1.58)	−0.2±0.79 (−2.02–1.08)	−0.91±1.01 (−3.91–1.58)**
% of impaired patients^1^	18	6	24
**FSMC total**	54±21 (20–99)	40±18 (20 – 87)	60±19 (20–90)***
% of pat. with mild fatigue (≥43)	13	10	14
% of pat. with moderate fatigue (≥53)	17	11	18
% of pat. with severe fatigue (≥63)	37	15	47

1 defined as performance falling below a critical z-score of 1.65.

2 Patients were considered to have cerebellar signs when the EDSS cerebellar functional systems score was >0.

Between group differences: *p≤0.05; **p≤0.01; ***p≤0.001.

Abbreviations: CIS = Clinically isolated syndrome suggestive of MS; RRMS = relapsing remitting MS; SPMS = secondary progressive MS; PPMS = primary progressive MS; EDSS = Expanded disability status scale; FSS = functional system score (EDSS subscore); 9-HPT = nine hole peg test; SDMT = symbol digit modalities test; PASAT = Paced Auditory Serial Addition Test; FSMC = Fatigue Scale for Motor and Cognitive Functions.

### Volumetric Measures

Normalized for brain size, and the raw data of the total cohort are listed in table 2.

**Table 2:** MRI data.

	Total cohort (n = 172)	Patients without cerebellarsigns (n = 52)	Patients with cerebellar signs^1^ (n = 120)
	mean ±SD (range)	mean ±SD (range)	mean ±SD (range)
**total T2 LV [cm^3^]**	6.1±6.6 (0–32.6)	3.5±4.0 (0–16.6)	7.2±7.2 (0–32.6)*
**cerebellar T1 LV [cm^3^]**	0.1±0.2 (0–1.4)	0.07±0.1 (0–0.5)	0.13±0.2 (0–0.8)
**raw data**			
Total brain volume [cm3]	1071±114 (857–1342)		
Total cerebellar volume [cm3]	137±14 (102–170)		
cerebellar GM volume [cm3]	113±11 (94–147)		
cerebellar WM volume [cm3]	25±5 (14–40)		
**normalized data**			
Total brain volume [cm3]	1428±98 (1136–1780)	1495±94 (1306–1780)	1400±85 (1136–1603)***
Total cerebellar volume [cm3]	183±18 (137–233)	191±17 (148–226)	180±17 (137–233)*
cerebellar GM volume [cm3]	151±15 (115–194)	155±14 (125–186)	148±15 (115–194)
cerebellar WM volume [cm3]	33±6 (20–47)	36±5 (24–47)	31±5 (20–45)***

1 Patients were considered to have cerebellar signs when the EDSS cerebellar functional systems score was >0**.** Between group differences: *p≤0.05; ***p≤0.001.

Abbreviations: T2 LV = T2 lesion volume; T1 LV = T1 lesion volume; GM = grey matter; WM = white matter.

### Group Effects

**Figure 1:**
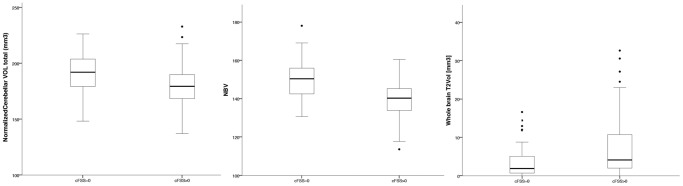
Between group differences. Box and whisker-plots of between-group differences of patients without (cFSS = 0; left column) and with cerebellar dysfunction (cFSS >0; right column). Plots are arranged as follows (from left to right): I) nTCV, II) NBV and III) whole brain T2 lesion volume (all volumes are shown in cm^3^). The boxplots display the median (bold line), the minimum (lower T-line) and maximum (upper T-line) as well as the first quartile (lower part of the box) and third quartile (upper part of the box) of shown volumes.

Significant between-group differences (ANCOVA) were observed between patients with and without cerebellar signs for several volumetric measurements (figure 1). This was true for nTCV (F(1, 168) = 4.98; p = 0.027), but not for cerebellar T1 LV. Age and gender had a significant effect on nTCV (age; F(1, 168) = 9.46; p = 0.002; gender: F(1, 168) = 4.16; p = 0.043). Moreover, in the case of nBV a significant group effect (F(1, 168) = 14.99, p<0.001), as well as an effect of age (F(1, 168) = 35.36, p<0.001) was observed. Further whole brain T2 LV differed significantly between groups (U = 2204; p = 0.003). A significant group effect (F(1, 167) = 39.77, p<0.001) and effect of gender (F(1, 167) = 11.85, p = 0.001) was observed for 9-HPT. Likewise, groups differed in SDMT (F(1, 146) = 17.89, p<0.001) and PASAT performance (F(1, 155) = 9.001, p = 0.003), as well as in FSMC-S score (F(1, 168) = 19.62, p<0.001).

The analysis was repeated dividing patients into three subgroups with increasing levels of cerebellar disability (cerebellar FSS 0 (n = 52), 1+2 (n = 77) or 3+4 (n = 43)). This showed a gradual reduction in both volumes and performance on cognitive tests. In this analysis, the group effect for TCV and cerebellar T1 LV was not statistically significant between the subgroups. The results of the remaining variables stayed unchanged.

### Relation of Brain Volumes to Cognitive Test Results (SDMT and PASAT)

**Figure 2:**
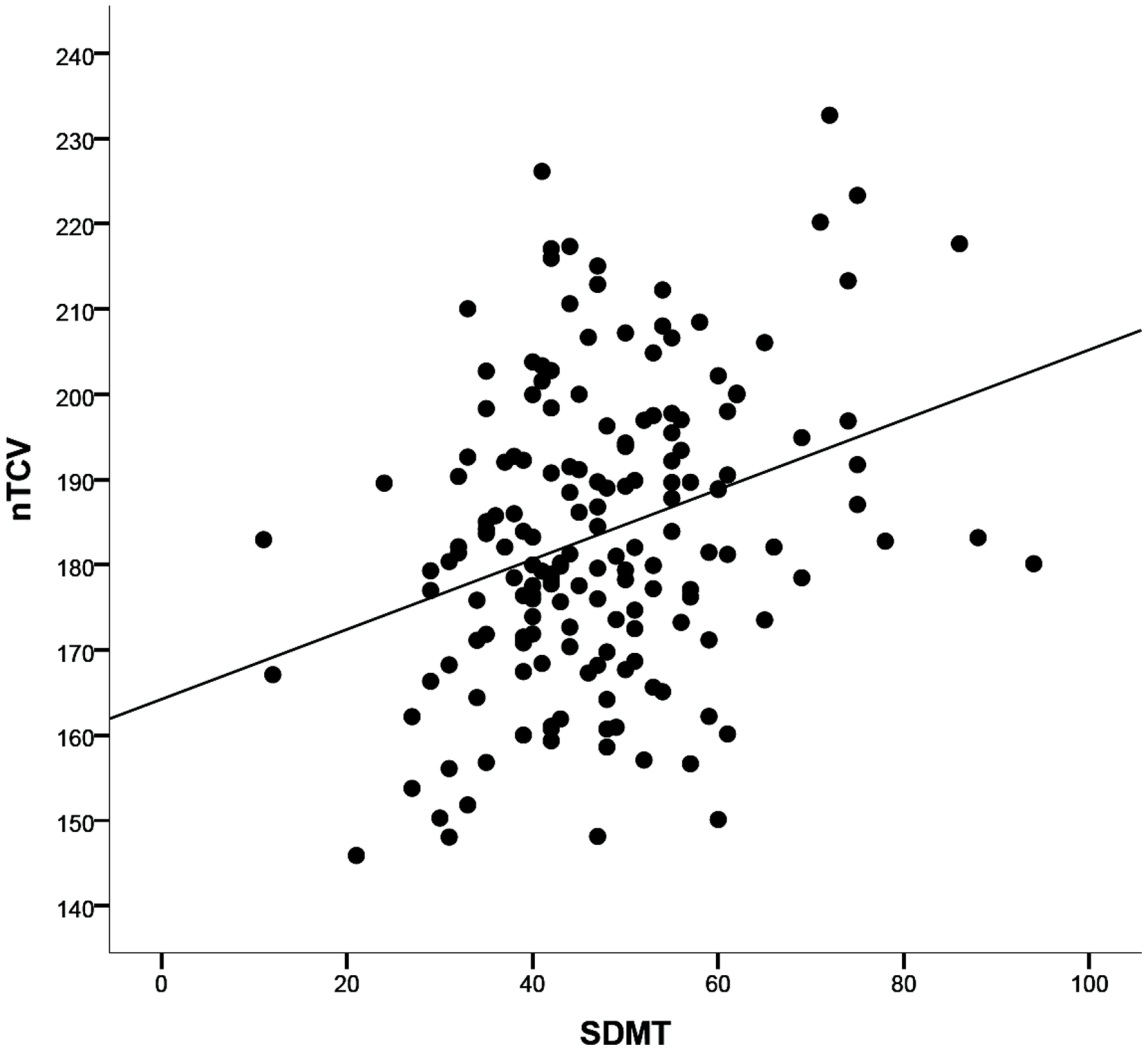
Relation between cerebellar volume and SDMT performance. Scatter plot showing the correlation between normalized total cerebellar volume (nTCV in cm3) vs SDMT performance (score).

In the first hierarchical MLR analysis, the relation between SDMT, cerebellar (nTCV, cerebellar T1 LV) and cerebral measures (nBV, whole brain T2 LV) was investigated. In the first regression block age and gender together explained 22% of SDMT variance (R^2^ = 0.22, F(2,164) = 22.54, p<0.001). The stepwise approach used in the second block identified nTCV (b = 0.19; F(3,163) = 17.80; p = 0.01) as a significant SDMT predictor (see figure 2). Twenty-five percent of the variance was explained in this block (R^2^ = 0.25, F(3,163) = 17.80, p = 0.01), which was significantly higher than in the first block (ΔR^2^ = 0.031, F-Change (1,163) = 6.74, p = 0.01). The full model (R^2^ = 0.36, F(5,161) = 17.90, p<0.001) showed that nBV (beta = 0.25, t(161) = 2.76, p = 0.007), together with age (beta = −0.28, t(161) =  −3.75, p<0.001) and whole brain T2 LV (beta = −0.208, t(161) = −2.86, p = 0.005) were independent predictors of SDMT.

A second hierarchical MLR analysis was identically designed to investigate the relation between PASAT scores, cerebellar volumes and cerebral measures. The stepwise approach identified cerebellar T1 LV (beta = 0.19, t(162) = 2.62, p = 0.01) as a significant predictor of PASAT scores in the second block. This together with age (beta = 0.35, t(162) = 4.86, p<0.001), explained 16% of the variance (R^2^ = 0.16, F(3,162) = 10.62, p<0.001). The full model (R^2^ = 0.23, F(4,161) = 12.06, p<0.001) showed that age (beta = 0.31, t(161) = 4.38, p<0.001) together with cerebellar T1 LV (beta = 0.14, t(161) = 2.01, p = 0.046) and whole brain T2 LV (beta = 0.27, t(161) = 3.72, p<0.001) were independent predictors of PASAT scores.

In the third hierarchical MLR analysis of FSMC-S score in relation to cerebellar and whole brain metrics (identical approach as for SDMT and PASAT) only age (beta = 0.294, t(167) = 3.57, p<0.001) and nBV (beta = −0.166, t(167) = −2.01, p = 0.046) emerged as significant predictors of the FMSC-S score, explaining 17% of the variance (R^2^ = 0.17, F(2,167) = 16.46, p<0.001) in the full model.

Subgroup analysis of only RRMS patients (n = 122) confirmed the results of the MLR models of the entire patient cohort. The significant predictors remained unchanged for SDMT and PASAT. In the case of FSMC-S, only age remained as a significant predictor.

## Discussion

We analysed cerebellar volumes in a large cohort of 172 MS patients and determined the relationship between cerebellar signs and cognitive performance.

The observed cerebellar volumes confirm previously reported numbers for MS patients, which are lower than healthy control data reported in the literature [16], [23]. We differentiated patients with and without cerebellar signs in the clinical examination and found that those patients with cerebellar signs had higher total EDSS scores, were older, and had longer disease durations.

We corrected for both age and gender due to their significant effect on nTCV. After correction, the total cerebellar volume was significantly reduced, with the lowest volumes in patients showing the highest degree of cerebellar disability, in comparison to the group of patients without cerebellar signs. Being aware of the technical difficulties of segmenting cerebellar white and grey matter, we decided to not include the segmentations of these separate compartments in any further between group or MLR analysis [17], [28].

We were especially interested in analysing to what extent the cognitive domains of information processing speed and working memory as well as fatigue, relate to cerebellar changes. Motor and cognitive fatigue are frequent and disabling symptoms which occur early in the disease course of MS [29]. Conventional imaging often fails to show a correlation of MRI measures and fatigue scores [30], [31]. More clear-cut results have been documented with functional imaging and studies of atrophy of selected brain regions. These studies evidenced that fatigue relates to changes in several cortical and subcortical areas [32], [33]. In our cohort, patients with cerebellar signs had a clearly higher FSMC-S score, than patients without cerebellar signs. However, in the MLR analysis, only age and nBV was predictive for FSMC-S and there was no significant association with cerebellar volumes. We, therefore, conclude that cerebellar pathology does not substantially contribute to the development of fatigue.

On the contrary, the detailed analysis of cognitive functions and their relation to cerebellar abnormalities provided further evidence that cerebellar signs and cognitive dysfunction occur in parallel. This confirms the results of anatomical and functional imaging studies [4], [7]. Patients with cerebellar signs performed significantly worse in SDMT and PASAT. The PASAT is a well-established tool for measuring deficits in working memory and processing speed in MS patients. PASAT has lost some popularity due to its dependency on arithmetic skills. Test subjects are easily frustrated and subsequently the reduced motivation may influence the test results. The SDMT has been suggested as an alternative assessment tool, suitable for testing information processing speed and working memory. In contrary to the PASAT, it is faster to conduct and easier to perform and therefore, more patient-friendly. However, direct comparisons of both tests in MS patients showed that SDMT scores are not necessarily predictive of PASAT scores [34]. Forn et al. have shown that during performance of both tests, a broad network of cortical (frontal, parietal and occipital), as well as, cerebellar regions are activated [35]. This suggests that SDMT and PASAT represent (partly) different cognitive domains. To better understand the relationship between cerebellar abnormalities and these cognitive tests in our cohort of MS patients, we calculated MLR models. In the case of SDMT, the total cerebellar volume explained part of the SDMT variance. This was not independent but rather dominated by wider spread MS related pathology in the brain as reflected by nBV and whole brain T2 LV. This is in accordance with previous studies [36]. Whereas, Yu et al. have described stronger associations of markers of cerebral and cerebellar white matter tract damage to the SDMT rather than PASAT [37], others have shown that widespread WM damage (including cerebellar peduncles) was correlated with lower PASAT performance [38]. However, none of these studies focused on the cerebellum. In our study, we have found that a poor PASAT performance seemed to be primarily related to cerebellar and whole brain lesion volumes rather than measures of atrophy.

Theoretically, results in patients with RRMS may differ from CIS or progressive MS, we therefore confirmed the MLR results in a subgroup analysis focusing only on RRMS patients. The significant predictors in this subgroup remained unchanged for SDMT and PASAT, as compared to the entire patient cohort. In the case of FSMC-S, nBV no longer reached statistical significance (p = 0.057) and only age remained as a significant predictor. Subgroup analysis of only CIS or only progressive patients was not feasible due to the low patient numbers in these groups.

In summary, cerebellar signs and cognitive symptoms are frequent and occur in parallel in MS patients. Our data support the view that cerebellar abnormalities contribute to cognitive impairment, whereas fatigue does not substantially relate to cerebellar abnormalities in MS. However, the role of the cerebellum is probably less dominant and not independent of wider spread diffuse pathology in other parts of the brain.
